# A case of spinal anesthesia in a patient with progressive supranuclear palsy

**DOI:** 10.1186/s40981-018-0149-2

**Published:** 2018-01-25

**Authors:** Momoka Tonan, Moritoki Egi, Nana Furushima, Satoshi Mizobuchi

**Affiliations:** 1 Department of Anesthesiology, Kobe University Hospital, 7-5-2, Kusunoki-cho, chuo-ku, Kobe, Hyogo 650-0017 Japan; 2 Division of Anesthesiology, Department of Surgery Related, Kobe University Graduate School of Medicine, 7-5-2, Kusunoki-cho, chuo-ku, Kobe, Hyogo 650-0017 Japan

**Keywords:** Progressive supranuclear palsy, Spinal anesthesia, Complication

## Abstract

Progressive supranuclear palsy (PSP) is one of the rare diseases. PSP is characterized by oculomotor dysfunction, postural instability, akinesia, dysarthria, and dysphagia. The major cause of death in patients with PSP is aspiration pneumonia. Considering these complications, spinal anesthesia is useful in patients with PSP. However, the potential harmful effects of spinal anesthesia including neurotoxicity of local anesthetics and neurologic complications for patients with PSP are unclear, because there has been no report. Here, we present spinal anesthesia for a patient with PSP. An 80-year-old man with progressive oculomotor dysfunction, dysphagia, and history of repeated aspiration pneumonia was scheduled for inguinal hernia surgery. Acutely concerning about perioperative pulmonary complications, we performed spinal anesthesia. Fortunately, there was no complication associated with respiration or neural system during perioperative period. We hope our experience and case report will be helpful in specific perioperative anesthetic care for patients with PSP.

## Background

Progressive supranuclear palsy (PSP) is a rare disease with a prevalence of 5.8–6.5 per 100,000 [1]. PSP is a progressing degenerative disease that is suspected to be related to tauopathies; however, its pathophysiology is still uncertain [2]. PSP is characterized by oculomotor dysfunction, postural instability, akinesia, dysarthria, and dysphagia [3]. It has been reported that the major cause of death in patients with PSP is aspiration pneumonia [4]. Considering the complications, spinal anesthesia is useful in patients with PSP. However, there is concern about the potential harmful effects of spinal anesthesia including neurotoxicity of local anesthetics and neurologic complications for patients with central nervous system disorder [5]. In this regard, the safety of spinal anesthesia for patients with PSP should be evaluated. However, there has been no report concerning spinal anesthesia in patients with PSP. Accordingly, we report a patient with PSP who had an operation for an inguinal hernia performed under spinal anesthesia.

## Case presentation

An 80-year-old man, 64 kg in weight and 166 cm in height, was scheduled for inguinal hernia surgery. He had been diagnosed with PSP 3 years before the operation based on progressive dysbasia, dysphagia and oculomotor dysfunction. Other medical history was hypertension requiring enalapril. He had received a partial pharyngectomy for middle pharynx cancer 5 years before the operation. His symptoms of PSP included weakness of coughing, mild cervical backward postural instability, dysphemia, and parkinsonism. He had mild cervical backward contracture. Dementia was not observed. Aspiration pneumonia had occurred once every few months, and his surgery had been repeatedly postponed. Suction of phlegm was needed every 2–3 h daily. Despite repeated aspiration pneumonia, preoperative chest radiography showed no consolidation, and the results of a respiratory function test showed 1.9 L of forced expiratory capacity (FEV1.0) and 79.6% of vital capacity (%VC). The results of electrocardiography and a blood examination were normal.

We decided to perform spinal anesthesia combined with epidural anesthesia for the patient. An epidural catheter was inserted 5 cm through Th12/L1. Spinal anesthesia was performed using 2.8 mL of 0.5% hyperbaric bupivacaine through L3/4. Surgery was started after confirming Th4 level of anesthesia by pinprick. During the surgery, the attending physician sucked secretion to eliminate oral retention in order to prevent aspiration pneumonia. The patient’s oxygen saturation was preserved at around 95% without any oxygen administration. The surgery was completed after 125 min without any complications. Swallowing function was checked 10 h later and oral intake was started on postoperative day 1 (POD 1). We confirmed that postoperative analgesia was sufficient only with acetaminophen administration on POD 1, and we stopped epidural anesthesia on POD 1. On POD 6, he was discharged from the hospital without aspiration pneumonia, exacerbation of neurological symptoms or any other complications.

## Discussion

For consideration of the anesthetic management for this case, we performed a literature search to find reports on anesthetic management in patients with PSP. We found seven case reports [6–12] (Table 1). There was no report on spinal anesthesia for a patient with PSP. Although the information in these reports and the results of our experiments are limited for drawing a conclusion, some anesthetic considerations could be obtained.

**Table 1:** Summary of case reports for anesthetic management in patients with progressive supranuclear palsy

Journal	Year	Age	Sex	Type of surgery	Maintenance anesthesia	Perioperative complications	Reference
J Anesth	2006	74	F	Laryngotracheal separation	General	Difficulty of awake intubation	[6]
Acta Anaesthesiol Taiwan	2012	81	F	Mammary tumor extirpation, ipsilateral lymph node excision	General	Difficulty of LMA fitting	[7]
Acta Anaesthesiol Taiwan.	2013	70	M	Above-knee amputation	General	–	[8]
J Clin Anesth	1987	72	F	Mammary tumor extirpation	Epidural	–	[9]
J Jpn Surg Assoc	1997	68	M	Sigmoid colon resection	Epidural + sevoflurane	Aagitation, needed nasal airway, abdominal rigidity during surgery	[10]
Jpn J Anesth	2010	60	F	Left cataract, right graucoma surgery	General	–	[11]
J Clin Anesth	2012	72	M	Gastrectomy	General + epidural	Delayed emergence, postoperative pneumonia	[12]

*M* male, *F* female

First, consideration should be given to the prevention of perioperative aspiration pneumonia. Takahashi et al. [11] reported that a patient with PSP after gastric surgery under general and epidural anesthesia had postoperative pneumonia. Progressive dysphasia and weakness of sputum excretion seen in patients with PSP would increase the risk of aspiration pneumonia. In this case, a history of partial pharyngectomy may worsen the symptoms of dysphasia in addition to those related to PSP. It has been reported that general anesthesia may increase the risk of postoperative pulmonary complications compared with regional anesthesia [13]. Arozullah et al. showed that the odds ratio of patients undergoing major non-cardiac surgery using general anesthesia was 1.56 (confidence interval, 1.36 to 1.80) compared with patients receiving local anesthesia [14]. Canet et al. [15] also reported that the risk for postoperative pulmonary complications with general anesthesia was 3.7-times higher than that with regional anesthesia. Thus, regional anesthesia seems to be effective for preventing aspiration pneumonia. However, it should be noted that regional anesthesia is relatively safe but not absolutely safe. Accordingly, we did not use any sedative agents and opiates, and secretion was sucked to eliminate oral retention during the operation.

Second, cervical dystonia may cause difficulties in airway management. Sakai et al. [6] have reported that awake orotracheal intubation was impossible for a patient with PSP because of strong airway stenosis caused by cervical backward tile and neck contracture. Maggi et al. [7] also reported difficulty in fitting on laryngeal airway mask in patients with PSP. Considering the awake orotracheal intubation reported by Sakai et al., it is possible that muscle relaxant was able to be used safety because sugamadex is available in Japan. Nonetheless, physicians should note the possibility of a difficult airway in patients with PSP.

The above two considerations related to the risk of pulmonary complications and management regional anesthesia may be preferable for patients with PSP, if appropriate. However, Lirk et al. [16] reported that regional anesthesia may be a risk for patients with pre-existing neuropathy such as diabetic peripheral neuropathy, multiple sclerosis, and Guillain-Barre syndrome. They suspected that spinal anesthesia may cause neuronal ischemia [17] or infarction owing to changes in endoneurial small vessels, blocking of sodium channels in demyelinated areas or interaction of regional anesthesia with peripheral myelin or nerve trauma, which may result in deterioration of symptoms. Accordingly, they suggested that spinal anesthesia should be avoided if there are safe alternatives in patients with pre-existing neuropathy. It should be noted that it is not clear whether spinal anesthesia for patients with PSP is safe since there has been no report for this scenario. In contrast to Lirk’s suggestion, Hebl et al. [18] reported that there were no new or deteriorating neurologic complications after neuraxial anesthesia, though technical complications occurred in 11% of the patients.

Our patient had considerable oral secretion and residual sputum with repeated aspiration pneumonia. He also had cervical backward contracture. Considering the balance of pulmonary and airway complications related to general anesthesia and the uncertain risk of progression of neural disorders, we decided to perform spinal anesthesia in our patient. We also inserted on epidural catheter to provide epidural anesthesia in case of prolonged surgery and for postoperative analgesia. The operation was performed without any trouble and the patient was discharged from hospital without any postoperative pneumonia or neurological complications. Nonetheless, taking into account the possibility of neurotoxicity of local anesthetics, a continuous peripheral nerve block with a percutaneously inserted catheter would be good alternative option for patients with PSP.

## Conclusions

In summary, we experienced spinal anesthesia for a patient with PSP. We hope our case report and experience will shed light on specific considerations in perioperative care for patients with PSP.

## Abbreviations

FEV: Forced expiratory capacity
POD: Postoperative day
PSP: Progressive supranuclear palsy
VC: Vital capacity
